# Body perception in chimpanzees and humans: The expert effect

**DOI:** 10.1038/s41598-020-63876-x

**Published:** 2020-04-28

**Authors:** Jie Gao, Fumito Kawakami, Masaki Tomonaga

**Affiliations:** 1 Primate Research Institute, Kyoto University, Inuyama, Aichi Japan; 2 Japan Society for the Promotion of Science, Tokyo, Japan; 3 Chubu University, Kasugai, Aichi Japan

**Keywords:** Psychology, Zoology

## Abstract

Both humans and chimpanzees have better performances when recognizing faces or bodies when the stimuli are upright compared to inverted. This is called the inversion effect. It suggests that these two species use a specific way to process faces and bodies. Previous research has suggested that humans also show the inversion effect to objects that they have expertise about, and this is called the expert effect. We investigated whether chimpanzees show the expert effect and how humans and chimpanzees differ by testing chimpanzees (human experts) with human body stimuli and testing humans (chimpanzee experts) with chimpanzee and human body stimuli in body recognition tasks. The main finding was that humans (chimpanzee experts) showed the expert effect to chimpanzee bodies, while chimpanzees partially showed it to human bodies. This suggests that compared with chimpanzees, the special processing in humans can be more flexibly tuned for other objects. We also tested humans that were not chimpanzee experts using chimpanzee body stimuli. Although they showed similar performances as the chimpanzee experts, the two groups had differences in some situations, indicating the effect of expertise. This study revealed the important role of experience in object processing in humans, and our evolutionary relatives, chimpanzees.

## Introduction

Faces and bodies provide very important social cues for animals. It has been widely reported that humans have a special way to process faces and bodies, as opposed to other objects, through configural processing (e.g., faces: ^1–4^; bodies: ^5–7^). Compared with other objects such as houses or pictures of scenery, the recognition of faces or bodies significantly deteriorates with inversion of the stimuli, which is called the inversion effect. The inversion effect of faces and bodies suggests that humans perceive them as a pre-set template and a holistic configuration rather than as combinations of local features, as is the case with other objects.

Humans’ perception narrows specifically to conspecifics^8^. Infants of 6 months old could discriminate faces of other species, while older infants and adults could not. However, despite the perception narrowing, many studies have found that the configural processing can be tuned for objects of expertise. In Diamond and Carey’s classic study^2^, it was found that dog experts showed the inversion effect when recognizing dogs, while dog novices did not. The researchers claimed that dogs, and all other biological classes whose members are individualized (in other words, having distinct bodies), can invoke configural processing, because they all share the same configuration. Dog experts are familiar with dogs, and are able to exploit the distinguishing internal relational features of dogs better than novices can, so they showed the expert effect and perceived dogs in the same way that people do with human faces (and bodies). This experiment has not always been successfully replicated^9,10^. However, there were several studies that showed the effect of expertise. Gauthier *et al*.^11^ reported that expertise for cars and birds shares brain areas with face recognition. Reed *et al*.^12^ also reported the effect of expertise on the configural processing of dogs by humans. Hagen and Tanaka^13^ reported that experts had partially shared neuro bases for within-category discrimination for human faces and birds, whereas novices did not. In a set of studies, when researchers trained novices to become experts about a series of artificial objects called “Greebles” that shared the same configuration but differed in internal relations, the participants showed face-like processing after acquiring the expertise^14–16^. These evidences suggest that humans are able to show configural processing for targets that they have expertise in, as with faces and bodies.

However, few studies have been done on other species’ abilities to show the expert effect (e.g., ^17^). One possible reason for this is that, in general, non-human animals do not have the need to distinguish other categories apart from the species themselves. Nevertheless, studying this ability in other species would shed light on the evolution of the expert effect in humans. It could help us to understand how humans are able to gain expertise, and how humans are able to tune for configural processing for quicker detection of stimuli that they have expertise with^18^.

Chimpanzees are humans’ closest relatives. They also show the inversion effect, i.e., configural processing to conspecifics’ faces^19–21^ and bodies^22^. Captive chimpanzees in research institutes see many people every day over the years, including caretakers, veterinarians, researchers, and visitors. They have intense interactions with many of them^23^. Therefore, they are a suitable group for testing for the presence and properties of the expert effect.

In our previous study^22^, we found that faces and body contours are the most important elements for evoking the body inversion effect in chimpanzees. For example, they did not show the inversion effect for bodies with blurred faces, but showed the inversion effect for bodies filled with mosaic patterns except for the faces (i.e., bodies with clear faces and blurred rest parts). They did not show the inversion effect for bodies without faces or for faces alone (of the same size as in the full body stimuli, which was very small in that experiment), but showed the inversion effect for chimpanzee silhouettes. However, processing of bodies of other species by chimpanzees has not been investigated. It is unclear whether or not they show the same properties of body configural processing to other species that they have expertise in. Regarding body processing by humans, previous research has revealed that the configural body processing in humans relies on the hierarchy of body parts, rather than the individual parts themselves^6^. Some studies found that the removal of the head would abolish or reverse the inversion effect, and thus the head is especially important^7,24^, while other studies found the inversion effect for headless bodies^25^ and argue that the head is not particularly important in body configural processing. Therefore, it is not clear how chimpanzees and humans differ in processing bodies of their conspecifics. Matsuno and Fujita^17^ studied how capuchin monkeys and humans process human bodies. Chimpanzees also show the inversion effect to humans’ faces^21^. However, there is a lack of studies involving direct comparison across species, which would facilitate greater understanding of the expert effect, and at the same time clarify the possible differences in the properties of the processing to the counter-species.

In this study, we used different kinds of human bodies to test chimpanzees at the Primate Research Institute who were human experts. We also used corresponding chimpanzee body stimuli to test humans who were chimpanzee experts. For comparison, we tested the chimpanzee experts with the same human body stimuli the chimpanzees received. Combing the results from our previous study^22^, where chimpanzees were tested using various kinds of chimpanzee bodies, there would be a 2×2 direct comparison matrix of human and chimpanzee participants processing human and chimpanzee bodies in order to understand the expert effect and detailed mechanisms of body processing in both species. We then did experiments with chimpanzee novices using the conditions of chimpanzee bodies where chimpanzee experts showed the inversion effect, in order to further investigate the expert effect in humans. In this study, we focused on body processing of bodies of these species in general, so the stimuli were all strange individuals to the participants to avoid any effect from familiarity.

## Results

### Humans as chimpanzee experts vs. chimpanzees as human experts

All “humans” mentioned in this part refer to chimpanzee experts.

#### Experiment 1 (participants: chimpanzees; stimuli: human bodies)

##### Summary

**Figure 1:**
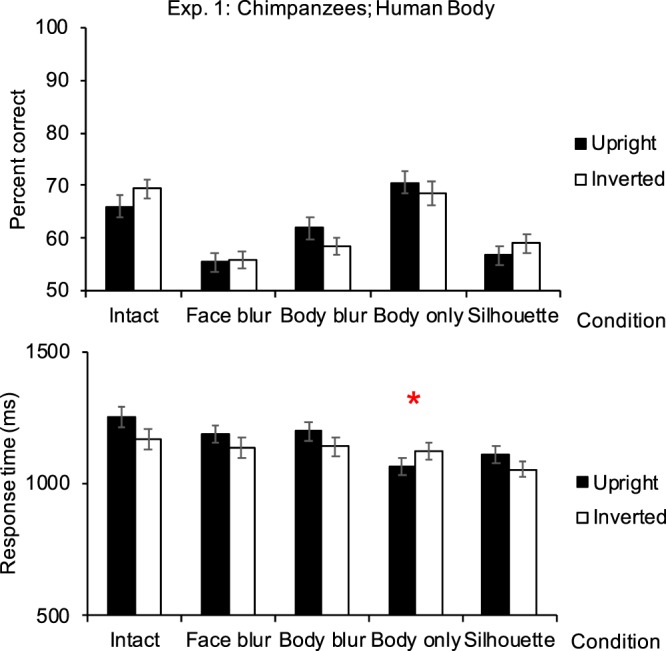
Performances in Experiment 1 (participants: chimpanzees, *N* = 7; stimuli: human bodies). Top figure shows the accuracy, and bottom figure shows the response time. Results showing positive inversion effect were marked with asterisks. Error bars: SEM; *p <0.05.

Chimpanzees only showed the inversion effect in the body-only condition (from response-time data, *p* = 0.026). No inversion effect was shown in the intact condition, the face-blur condition, the body-blur condition, or the silhouette condition (Fig. 1; Table 1).

**Table 1:** The inversion effect in each condition of the four experiments.

Stimuli	Condition	Chimpanzees (human experts) were participants	Humans (chimpanzee experts) were participants
Chimpanzee bodies	Intact	+	+
	Face blur	−	+
	Body blur	+	+
	Body only	−	+
	Silhouette	+	−
Human bodies	Intact	−*	+
	Face blur	−	+
	Body blur	−*	+
	Body only	+	+
	Silhouette	−*	+

Note: “+” indicates the inversion effect in the condition, and “−” indicates no inversion effect. The data of chimpanzee participants with chimpanzee body stimuli is from our previous study^22^.

*significant in inverted inversion effect (Error rates or response times were significantly higher in the upright trials than those in the inverted trials).

##### Accuracy

In the intact body condition, the mean accuracy in the upright trials was 66.071 ± 2.228%, and the mean accuracy in the inverted trials was 69.464 ± 1.809%. There was no difference between the upright and inverted trials (estimate of the fixed effect orientation: −0.165, standard error: 0.093, *z* value = −1.768, *p* = 0.077; estimate of the intercept: 0.890). The variances of the random effects, participant ID and session number were 0.301 and 0.046, respectively, and their standard deviations (*SD*s) were 0.549 and 0.215, respectively. In the face-blur condition, the mean accuracy in the upright trials was 55.536 ± 1.818%, and the mean accuracy in the inverted trials was 55.804 ± 1.650%. There was no difference between the upright and inverted trials (estimate of the fixed effect orientation: −0.011, standard error: 0.086, *z* value = −0.129, *p* = 0.897; estimate of the intercept: 0.239). The variances of the random effects, participant ID and session number were 0.087 and 0.012, respectively, and their *SD*s were 0.294 and 0.108, respectively. In the body-blur condition, the mean accuracy in the upright trials was 61.964 ± 2.142%, and the mean accuracy in the inverted trials was 58.571 ± 1.569%. There was no difference between the upright and inverted trials (estimate of the fixed effect orientation: 0.147, standard error: 0.088, *z* value = 1.672, *p* = 0.095; estimate of the intercept: 0.365). The variances of the random effects, participant ID and session number were 0.161 and 0.021, respectively, and their *SD*s were 0.401 and 0.145, respectively. In the body-only condition, the mean accuracy in the upright trials was 70.625 ± 2.043%, and the mean accuracy in the inverted trials was 68.571 ± 2.242%. There was no difference between the upright and inverted trials (estimate of the fixed effect orientation: 0.102, standard error: 0.094, *z* value = 1.083, *p* = 0.279; estimate of the intercept: 0.827). The variances of the random effects, participant ID and session number were 0.167 and 0.102, respectively, and their *SD*s were 0.409 and 0.319, respectively. In the silhouette condition, the mean accuracy in the upright trials was 56.786 ± 1.835%, and the mean accuracy in the inverted trials was 59.018 ± 1.866%. There was no difference between the upright and inverted trials (estimate of the fixed effect orientation: −0.094, standard error: 0.087, *z* value = −1.085, *p* = 0.278; estimate of the intercept: 0.374). The variances of the random effects, participant ID and session number were 0.118 and <0.001, respectively, and their *SD*s were 0.344 and <0.001, respectively.

##### Response time

In the intact body condition, the mean response time in the upright trials was 1254 ± 37 ms, and the mean response time in the inverted trials was 1170 ± 40 ms. The response time was significantly higher in the upright trials than that in the inverted trials (estimate of the fixed effect orientation: > −0.001, standard error: <0.001, *t* value = −4.726, *p* <0.001; estimate of the intercept: <0.001). The variances and *SD*s of the random effects, participant ID and session number, were all <0.001. In the face-blur condition, the mean response time in the upright trials was 1190 ± 33 ms, and the mean response time in the inverted trials was 1136 ± 37 ms. There was no difference between the upright and inverted trials (estimate of the fixed effect orientation: > −0.001, standard error: <0.001, *t* value = −1.804, *p* = 0.071; estimate of the intercept: <0.001). The variances and *SD*s of the random effects, participant ID and session number, were all <0.001. In the body-blur condition, the mean response time in the upright trials was 1200 ± 35 ms, and the mean response time in the inverted trials was 1141 ± 33 ms. The response time in the upright trials were slightly higher than that in the inverted trials (estimate of the fixed effect orientation: >−0.001, standard error: <0.001, *t* value = −1.896, *p* = 0.058; estimate of the intercept: <0.001). The variances and SDs of the random effects, participant ID and session number, were all <0.001. In the body-only condition, the mean response time in the upright trials was 1065 ± 32 ms, and the mean response time inthe inverted trials was 1124 ± 35 ms. The response time in the inverted trials were significantly higher than that in the upright trials (estimate of the fixed effect orientation: <0.001, standard error: <0.001, *t* value = 2.223, *p* = 0.026; estimate of the intercept: <0.001). The variances and *SD*s of the random effects, participant ID and session number, were all <0.001. In the silhouette condition, the mean response time in the upright trials was 1111 ± 32 ms, and the mean response time in the inverted trials was 1055 ± 30 ms. The response time in the upright trials were significantly higher than that in the inverted trials (estimate of the fixed effect orientation: > −0.001, standard error: <0.001, *t* value = −2.010, *p* = 0.045; estimate of the intercept: <0.001). The variances and *SD*s of the random effects, participant ID and session number, were all <0.001.

#### Experiment 2 (participants: humans [chimpanzee experts]; stimuli: chimpanzee bodies; 8 out the 9 participants in this experiment were the participants in Experiment 3)

##### Summary

**Figure 2:**
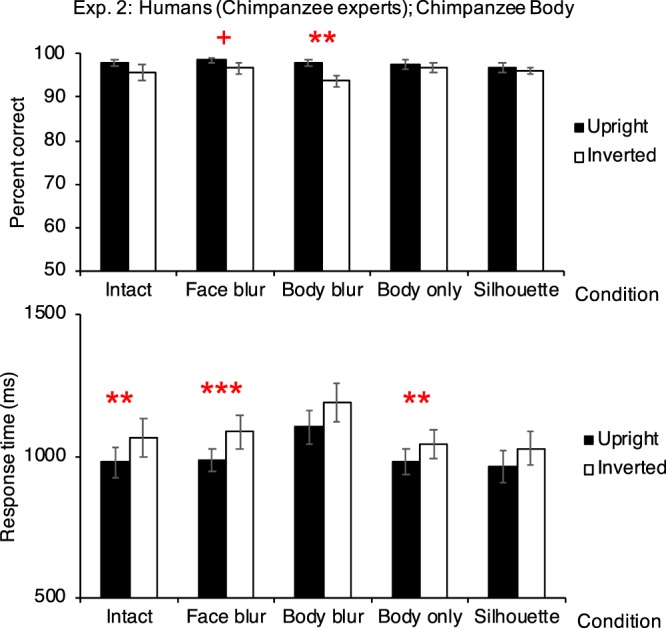
Performances in Experiment 2 (participants: humans [chimpanzee experts], *N* = 9; stimuli: chimpanzee bodies). Top figure shows the accuracy, and bottom figure shows the response time. Results showing positive inversion effect were marked. Error bars: SEM; + *p* <0.06; **p <0.01; ***p <0.001.

The human participants showed the inversion effect in the intact condition (from response-time data, *p* = 0.008), the face-blur condition (from accuracy and response-time data, *p* = 0.051 and <0.001 respectively), the body-blur condition (accuracy, *p* = 0.001), and the body-only condition (response-time, *p* = 0.002). No inversion effect was shown in the silhouette condition (Fig. 2; Table 1).

##### Accuracy

In the intact body condition, the mean accuracy in the upright trials was 97.778 ± 0.680%, and the mean accuracy in the inverted trials was 95.741 ± 1.824%. There was no significant difference between the accuracy in the upright and inverted trials (estimate of the fixed effect orientation: 0.678, standard error: 0.363, *z* value = 1.867, *p* = 0.062; estimate of the intercept: 3.243). The variance and *SD* of the random effect, participant ID, were 0.290 and 0.538, respectively. In the face-blur condition, the mean accuracy in the upright trials was 98.519 ± 0.516%, and the mean accuracy in the inverted trials was 96.667 ± 1.303%. The accuracy in the upright trials were (marginally) significantly higher than that in the inverted trials (estimate of the fixed effect orientation: 0.846, standard error: 0.433, *z* value = 1.953, *p* = 0.051; estimate of the intercept: 3.833). The variance and *SD* of the random effect, participant ID, were 1.151 and 1.073, respectively. In the body-blur condition, the mean accuracy in the upright trials was 97.778 ± 0.735%, and the mean accuracy in the inverted trials was 93.704 ± 1.296%. The accuracy in the upright trials were significantly higher than that in the inverted trials (estimate of the fixed effect orientation: 1.088, standard error: 0.342, *z* value = 3.181, *p* = 0.001; estimate of the intercept: 2.738). The variance and *SD* of the random effect, participant ID, were 0.185 and 0.430, respectively. In the body-only condition, the mean accuracy in the upright trials was 97.407 ± 1.043%, and the mean accuracy in the inverted trials was 96.852 ± 1.056%. No difference was found between the upright and inverted trials (estimate of the fixed effect orientation: 0.204, standard error: 0.370, *z* value = 0.552, *p* = 0.581; estimate of the intercept: 3.803). The variance and *SD* of the random effect, participant ID, were 0.898 and 0.948, respectively. In the silhouette condition, the mean accuracy in the upright trials was 96.852 ± 1.160%, and the mean accuracy in the inverted trials was 96.111 ± 0.833%. No difference was found between the upri ght and inverted trials (estimate of the fixed effect orientation: 0.222, standard error: 0.334, *z* value = 0.664, *p* = 0.507; estimate of the intercept: 3.387). The variance and *SD* of the random effect, participant ID, were 0.408 and 0.639, respectively.

##### Response time

In the intact body condition, the mean response time in the upright trials was 980 ± 54 ms, and the mean response time in the inverted trials was 1067 ± 66 ms. The response time in the inverted trials were significantly higher than that in the upright trials (estimate of the fixed effect orientation: <0.001, standard error: <0.001, *t* value = 2.649, *p* = 0.008; estimate of the intercept: <0.001). The variance and *SD* of the random effect, participant ID, were both <0.001. In the face-blur condition, the mean response time in the upright trials was 985 ± 40 ms, and the mean response time in the inverted trials was 1086 ± 61 ms. The response time in the inverted trials were significantly higher than that in the upright trials (estimate of the fixed effect orientation: <0.001, standard error: <0.001, *t* value = 3.351, *p* <0.001; estimate of the intercept: <0.001). The variance and *SD* of the random effect, participant ID, were both <0.001. In the body-blur condition, the mean response time in the upright trials was 1103 ± 60 ms, and the mean response time in the inverted trials was 1189 ± 67 ms. No significant difference was found between the upright and inverted trials (estimate of the fixed effect orientation: <0.001, standard error: <0.001, *t* value = 1.783, *p* = 0.075; estimate of the intercept: <0.001). The variance and *SD* of the random effect, participant ID, were both <0.001. In the body-only condition, the mean response time in the upright trials was 980 ± 44 ms, and the mean response time in the inverted trials was 1043 ± 52 ms. The response time in the inverted trials were significantly higher than that in the upright trials (estimate of the fixed effect orientation: <0.001, standard error: <0.001, *t* value = 3.063, *p* = 0.002; estimate of the intercept: <0.001). The variance and *SD* of the random effect, participant ID, were both <0.001. In the silhouette condition, the mean response time in the upright trials was 965 ± 55 ms, and the mean response time in the inverted trials was 1028 ± 59 ms. No significant difference was found between the upright and inverted trials (estimate of the fixed effect orientation: <0.001, standard error: <0.001, *t* value = 1.604, *p* = 0.109; estimate of the intercept: <0.001). The variance and *SD* of the random effect, participant ID, were both <0.001.

#### Experiment 3 (participants: humans [chimpazee experts]; stimuli: human bodies)

##### Summary

**Figure 3:**
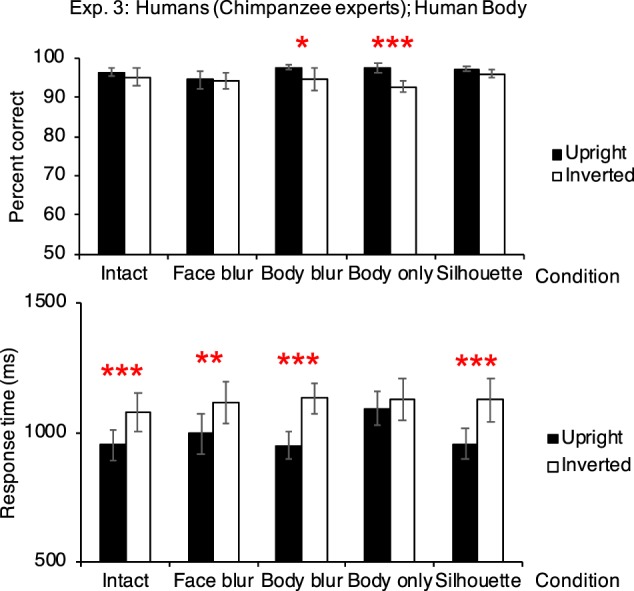
Performances in Experiment 3 (participants: humans [chimpanzee experts], *N* = 8; stimuli: human bodies). Top figure shows the accuracy, and bottom figure shows the response time. Results showing positive inversion effect were marked with asterisks. Error bars: SEM; *p <0.05; **p <0.01; ***p <0.001.

Participants showed the inversion effect in all the conditions: the intact condition (from response-time data, *p* <0.001), the face-blur condition (response-time, *p* = 0.002), the body-blur condition (accuracy and response-time, *p* = 0.018 and <0.001 respectively), the body-only condition (accuracy, *p* <0.001), and the silhouette condition (response-time, *p* <0.001; Fig. 3; Table 1).

##### Accuracy

In the intact body condition, the mean accuracy in the upright trials was 96.458 ± 1.111%, and the mean accuracy in the inverted trials was 95.208 ± 2.281%. No difference was found between the upright and inverted trials (estimate of the fixed effect orientation: 0.327, standard error: 0.333, *z* value = 0.984, *p* = 0.325; estimate of the intercept: 3.392). The variance and *SD* of the random effect, participant ID, were 0.903 and 0.950, respectively. In the face-blur condition, the mean accuracy in the upright trials was 94.583 ± 2.266%, and the mean accuracy in the inverted trials was 94.167 ± 1.916%. No difference was found between the upright and inverted trials (estimate of the fixed effect orientation: 0.082, standard error: 0.282, *z* value = 0.290, *p* = 0.772; estimate of the intercept: 3.012). The variance and *SD* of the random effect, participant ID, were 0.471 and 0.687, respectively. In the body-blur condition, the mean accuracy in the upright trials was 97.708 ± 0.700%, and the mean accuracy in the inverted trials was 94.792 ± 2.894%. The accuracy in the upright trials were significantly higher than that in the inverted trials (estimate of the fixed effect orientation: 0.883, standard error: 0.374, *z* value = 2.360, *p* = 0.018; estimate of the intercept: 3.251). The variance and *SD* of the random effect, participant ID, were 0.737 and 0.859, respectively. In the body-only condition, the mean accuracy in the upright trials was 97.500 ± 1.136%, and the mean accuracy in the inverted trials was 92.708 ± 1.371%. The accuracy in the upright trials were significantly higher than that in the inverted trials (estimate of the fixed effect orientation: 1.125, standard error: 0.340, *z* value = 3.304, *p* <0.001; estimate of the intercept: 2.577). The variance and *SD* of the random effect, participant ID, were 0.081 and 0.285, respectively. In the silhouette condition, the mean accuracy in the upright trials was 97.292 ± 0.625%, and the mean accuracy in the inverted trials was 96.042 ± 0.942%. No difference was found between the upright and inverted trials (estimate of the fixed effect orientation: 0.393, standard error: 0.366, *z* value = 1.073, *p* = 0.283; estimate of the intercept: 3.194). The variance and *SD* of the random effect, participant ID, were 0.010 and 0.102, respectively.

##### Response time

In the intact body condition, the mean response time in the upright trials was 952 ± 58 ms, and the mea response time in the inverted trials was 1080 ± 74 ms. The response time in the inverted trials were significantly higher than that in the upright trials (estimate of the fixed effect orientation: <0.001, standard error: <0.001, *t* value = 4.753, *p* <0.001; estimate of the intercept: <0.001). The variance and *SD* of the random effect, participant ID, were both <0.001. In the face-blur condition, the mean response time in the upright trials was 995 ± 76 ms, and the mean response time in the inverted trials was 1115 ± 83 ms. The response time in the inverted trials were significantly higher than that in the upright trials (estimate of the fixed effect orientation: <0.001, standard error: <0.001, *t* value = 3.159, *p* = 0.002; estimate of the intercept: <0.001). The variance and *SD* of the random effect, participant ID, were both <0.001. In the body-blur condition, the mean response time in the upright trials was 950 ± 51 ms, and the mean response time in the inverted trials was 1133 ± 59 ms. The response time in the inverted trials were significantly higher than that in the upright trials (estimate of the fixed effect orientation: <0.001, standard error: <0.001, *t* value = 5.983, *p* <0.001; estimate of the intercept: <0.001). The variance and *SD* of the random effect, participant ID, were both <0.001. In the body-only condition, the mean response time in the upright trials was 1093 ± 65 ms, and the mean response time in the inverted trials was 1128 ± 81 ms. No significant difference was found between the upright and inverted trials (estimate of the fixed effect orientation: <0.001, standard error: <0.001, *t* value = 1.193, *p* = 0.233; estimate of the intercept: <0.001). The variance and *SD* of the random effect, participant ID, were both <0.001. In the silhouette condition, the mean response time in the upright trials was 956 ± 59 ms, and the mean response time in the inverted trials was 1125 ± 86 ms. The response time in the inverted trials were significantly higher than that in the upright trials (estimate of the fixed effect orientation: <0.001, standard error: <0.001, *t* value = 5.178, *p* <0.001; estimate of the intercept: <0.001). The variance and *SD* of the random effect, participant ID, were both <0.001.

#### Previous study (participants: chimpanzees; stimuli: chimpanzee bodies)

The chimpanzees showed the inversion effect in the intact condition (from accuracy data), the body-blur condition (accuracy), and the silhouette condition (accuracy). No inversion effect was shown in the face-blur condition or the body-only condition (Table 1; See also 22).

### Chimpanzee experts (human participants) vs. chimpanzee novices (human participants)

#### Experiment 4 (participants: humans [chimpanzee novices]; stimuli: chimpanzee bodies)

Only the conditions of chimpanzee bodies where the chimpanzee experts showed the inversion effect were tested, i.e., intact body condition, face-blur condition, body-blur condition, and body-only condition.

##### Summary

**Figure 4:**
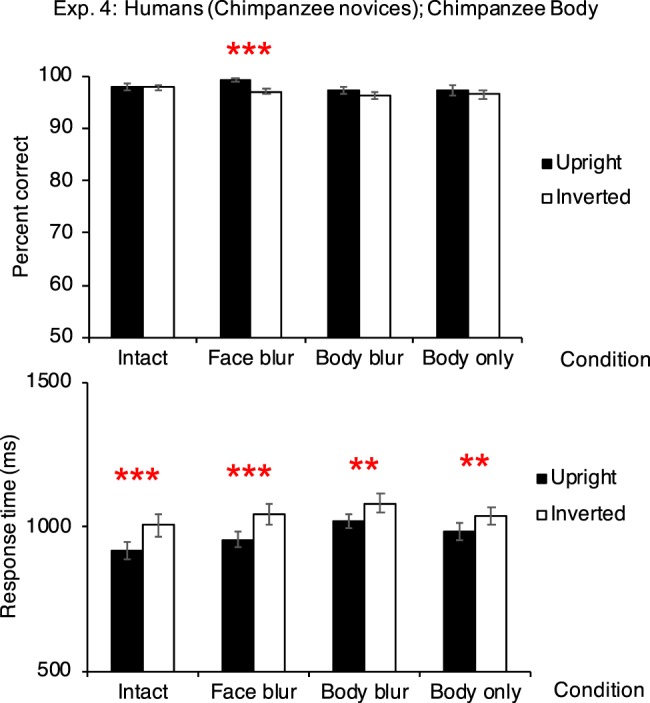
Performances in Experiment 4 (participants: humans [chimpanzee novices], *N* = 20; stimuli: chimpanzee bodies). Top figure shows the accuracy, and bottom figure shows the response time. Results showing positive inversion effect were marked. Error bars: SEM; **p <0.01; ***p <0.001.

The novice human participants showed the inversion effect in the intact condition (from response-time data, *p* <0.001), the face-blur condition (from both accuracy and response-time data, both *p* values <0.001), the body-blur condition (response-time, *p* = 0.002), and the body-only condition (response-time, *p* = 0.008; Fig. 4).

##### Accuracy

In the intact body condition, the mean accuracy in the upright trials was 98.000 ± 0.600%, and the mean accuracy in the inverted trials was 97.833 ± 0.365%. The accuracy in the upright trials and in the inverted trials did not differ significantly (estimate of the fixed effect orientation: 0.082, standard error: 0.287, *z* value = 0.287, *p* = 0.774; estimate of the intercept: 3.949). The variance and *SD* of the random effect, participant ID, were 0.295 and 0.543, respectively. In the face-blur condition, the mean accuracy in the upright trials was 99.167 ± 0.331%, and the mean accuracy in the inverted trials was 97.083 ± 0.578%. The accuracy in the upright trials were significantly higher than that in the inverted trials (estimate of the fixed effect orientation: 1.286, standard error: 0.362, *z* value = 3.549, *p* <0.001; estimate of the intercept: 3.767). The variance and *SD* of the random effect, participant ID, were 0.581 and 0.762, respectively. In the body-blur condition, the mean accuracy in the upright trials was 97.333 ± 0.621%, and the mean accuracy in the inverted trials was 96.167 ± 0.663%. There was no significant difference in accuracy in the upright and inverted trials (estimate of the fixed effect orientation: 0.380, standard error: 0.236, *z* value = 1.614, *p* = 0.106; estimate of the intercept: 3.411). The variance and *SD* of the random effect, participant ID, were 0.416 and 0.645, respectively. In the body-only condition, the mean accuracy in the upright trials was 97.250 ± 0.939%, and the mean accuracy in the inverted trials was 96.500 ± 0.801%. No difference was found between the upright and inverted trials (estimate of the fixed effect orientation: 0.256, standard error: 0.239, *z* value = 1.067, *p* = 0.286; estimate of the intercept: 3.692). The variance and *SD* of the random effect, participant ID, were 0.844 and 0.919, respectively.

##### Response time

In the intact body condition, the mean response time in the upright trials was 916 ± 30 ms, and the mean response time in the inverted trials was 1006 ± 39 ms. The response time in the inverted trials were significantly higher than that in the upright trials (estimate of the fixed effect orientation: <0.001, standard error: <0.001, *t* value = 5.32, *p* <0.001; estimate of the intercept: = 0.001). The variance and *SD* of the random effect, participant ID, were both <0.001. In the face-blur condition, the mean response time in the upright trials was 957 ± 27 ms, and the mean response time in the inverted trials was 1044 ± 36 ms. The response time in the inverted trials were significantly higher than that in the upright trials (estimate of the fixed effect orientation: <0.001, standard error: <0.001, *t* value = 5.48, *p* <0.001; estimate of the intercept: <0.001). The variance and *SD* of the random effect, participant ID, were both <0.001. In the body-blur condition, the mean response time in the upright trials was 1020 ± 24 ms, and the mean response time in the inverted trials was 1083 ± 34 ms. The response time in the inverted trials were significantly higher than that in the upright trials (estimate of the fixed effect orientation: <0.001, standard error: <0.001, *t* value = 3.13, *p* = 0.002; estimate of the intercept: <0.001). The variance and *SD* of the random effect, participant ID, were both <0.001. In the body-only condition, the mean response time in the upright trials was 987 ± 30 ms, and the mean response time in the inverted trials was 1040 ± 28 ms. The response time in the inverted trials were significantly higher than that in the upright trials (estimate of the fixed effect orientation: <0.001, standard error: <0.001, *t* value = 2.67, *p* = 0.008; estimate of the intercept: <0.001). The variance and *SD* of the random effect, participant ID, were both <0.001.

#### Comparison of results of chimpanzee experts and novices

##### Summary

There was no significant interaction between orientation (upright or inverted) and group (experts or novices) in all the conditions. Novices showed marginally significantly higher accuracies than experts in the inverted trials of the intact condition (accuracy data, *p* = 0.057). Their performances were not significantly different in the inverted trials of other conditions, or upright trials of all the conditions.

##### Accuracy

In the intact condition, the performance of two groups (experts and novices) did not differ in upright trials (estimate of the fixed effect group: −0.154, standard error: 0.447, *z* value = −0.345, *p* = 0.730; estimate of the intercept: 4.031; estimate of the fixed effect orientation: −0.082; estimate of the fixed effect, interaction between orientation and group: −0.595). However, the accuracy of novices was marginally significantly higher than experts in inverted trials (estimate of the fixed effect group: −0.750, standard error: 0.394, *z* value = −1.902, *p* = 0.057; estimate of the intercept: 3.949; estimate of the fixed effect orientation: 0.082; estimate of the fixed effect, interaction between orientation and group: 0.595). The interaction was not significant (*p* = 0.198).

In the face-blur condition, the performance of two groups did not differ in upright trials (estimate of the fixed effect group: −0.511, standard error: 0.660, *z* value = −0.774, *p* = 0.439; estimate of the intercept: 4.926; estimate of the fixed effect orientation: −1.281; estimate of the fixed effect, interaction between orientation and group: 0.439) or inverted trials (estimate of the fixed effect group: −0.072, standard error: 0.542, *z* value = −0.134, *p* = 0.894; estimate of the intercept: 3.646; estimate of the fixed effect orientation: 1.281; estimate of the fixed effect, interaction between orientation and group: −0.439). The interaction was not significant (*p* = 0.436).

In the body-blur condition, the performance of two groups did not differ in upright trials (estimate of the fixed effect group: 0.075, standard error: 0.432, *z* value = 0.174, *p* = 0.862; estimate of the intercept: 3.789; estimate of the fixed effect orientation: −0.380; estimate of the fixed effect, interaction between orientation and group: −0.712) or inverted trials (estimate of the fixed effect group: −0.637, standard error: 0.349, *z* value = −1.824, *p* = 0.068; estimate of the intercept: 3.409; estimate of the fixed effect orientation: 0.380; estimate of the fixed effect, interaction between orientation and group: 0.712). The interaction was not significant (*p* = 0.087).

In the body-only condition, the performance of two groups did not differ in upright trials (estimate of the fixed effect group: 0.271, standard error: 0.683, *z* value = 0.397, *p* = 0.691; estimate of the intercept: 3.932; estimate of the fixed effect orientation: −0.256; estimate of the fixed effect, interaction between orientation and group: 0.051) or inverted trials (estimate of the fixed effect group: 0.323, standard error: 0.667, *z* value = 0.484, *p* = 0.629; estimate of the intercept: 3.676; estimate of the fixed effect orientation: 0.256; estimate of the fixed effect, interaction between orientation and group: −0.051). The interaction was not significant (*p* = 0.907).

##### Response time

In the intact condition, the performance of two groups did not differ in upright trials (estimate of the fixed effect group: > −0.001, standard error: <0.001, *t* value = −1.14, *p* = 0.256; estimate of the intercept: 0.001; estimate of the fixed effect orientation: > −0.001; estimate of the fixed effect, interaction between orientation and group: <0.001) or inverted trials (estimate of the fixed effect group: > −0.001, standard error: <0.001, *t* value = −0.89, *p* = 0.375; estimate of the intercept: 0.001; estimate of the fixed effect orientation: <0.001; estimate of the fixed effect, interaction between orientation and group: > −0.001). The interaction was not significant (*p* = 0.659).

In the face-blur condition, the performance of two groups did not differ in upright trials (estimate of the fixed effect group: > −0.001, standard error: <0.001, *t* value = −0.79, *p* = 0.431; estimate of the intercept: 0.001; estimate of the fixed effect orientation: > −0.001; estimate of the fixed effect, interaction between orientation and group: > −0.001) or inverted trials (estimate of the fixed effect group: > −0.001, standard error: <0.001, *t* value = −0.96, *p* = 0.335; estimate of the intercept: <0.001; estimate of the fixed effect orientation: <0.001; estimate of the fixed effect, interaction between orientation and group: <0.001). The interaction was not significant (*p* = 0.823).

In the body-blur condition, the performance of two groups did not differ in upright trials (estimate of the fixed effect group: > −0.001, standard error: <0.001, *t* value = −1.42, *p* = 0.157; estimate of the intercept: <0.001; estimate of the fixed effect orientation: > −0.001; estimate of the fixed effect, interaction between orientation and group: > −0.001) or inverted trials (estimate of the fixed effect group: > −0.001, standard error: <0.001, *t* value = −1.63, *p* = 0.104; estimate of the intercept: <0.001; estimate of the fixed effect orientation: <0.001; estimate of the fixed effect, interaction between orientation and group: <0.001). The interaction was not significant (*p* = 0.813).

In the body-only condition, the performance of two groups did not differ in upright trials (estimate of the fixed effect group: <0.001, standard error: <0.001, *t* value = 0.31, *p* = 0.758; estimate of the intercept: <0.001; estimate of the fixed effect orientation: > −0.001; estimate of the fixed effect, interaction between orientation and group: > −0.001) or inverted trials (estimate of the fixed effect group: <0.001, standard error: <0.001, *t* value = 0.07, *p* = 0.943; estimate of the intercept: <0.001; estimate of the fixed effect orientation: <0.001; estimate of the fixed effect, interaction between orientation and group: <0.001). The interaction was not significant (*p* = 0.735).

### Control stimuli, houses, for human participants

In this paper, we did not include any condition of stimuli of other objects other than bodies for control in the experiments with chimpanzee participants. The same chimpanzee participants have been tested with stimuli of houses in the same matching-to-sample paradigm in a previous study^22^. The chimpanzees were familiar with houses, but they did not show any inversion effect. Like the case with chimpanzee participants, to confirm the inversion effects by human participants in this study are valid to show configural processing, which is different from the featural processing used for other objects, we tested human participants using stimuli of house pictures.

The participants were the same as in Experiment 2 (chimpanzee experts; *N* = 9). The procedure was the same as Experiment 2 and 3, and the only difference was the stimuli. They did not show significant inversion effect in this experiment. The mean accuracy in the upright trials was 98.888 ± 0.393%, and the mean accuracy in the inverted trials was 98.296 ± 0.736%. The accuracy in the upright trials and in the inverted trials did not differ significantly (estimate of the fixed effect orientation: 0.414, standard error: 0.532, *z* value = 0.777, *p* = 0.437; estimate of the intercept: 4.321). The variance and *SD* of the random effect, participant ID, were 0.521 and 0.722, respectively. The mean response time in the upright trials was 856 ± 35 ms, and the mean response time in the inverted trials was 889 ± 32 ms. The response time in the upright trials and in the inverted trials did not differ significantly (estimate of the fixed effect orientation: <0.001, standard error: <0.001, *t* value = 1.84, *p* = 0.066; estimate of the intercept: = 0.001). The variance and *SD* of the random effect, participant ID, were both <0.001.

## Discussions

In this study, we tested chimpanzees using human body stimuli and humans (chimpanzee experts) using human and chimpanzee body stimuli to examine their ways of processing conspecifics and the other species. We found that humans showed the expert effect to chimpanzees, while chimpanzees partially showed that to humans. We also tested humans who were chimpanzee novices using chimpanzee body stimuli, which the chimpanzee experts showed the inversion effect to, and found that there was no significant difference beteen the experts and novices.

However, there was a marginally significant difference between the experts and novices in the intact chimpanzee body condition. The novices showed higher accuracy than experts in the inverted orientation (*p* = 0.057). It is consistent with the participants’ level of expertise about chimpanzees. The experts, compared to novices, are more tuned to use configural processing with chimpanzee stimuli. They tend to process chimpanzee bodies as a fixed configuration more than novices, thus showing poorer recognition when the chimpanzee bodies were inverted. Although this difference in the inverted orientation did not lead to a significant difference in the amplitude of the inversion effect in this intact chimpanzee body condition, there was a tendency that the experts and novices had different levels of inversion effect in the body-blur condition. In the inverted orientation of the body-blur condition (with chimpanzee stimuli), the novices, too, showed higher accuracy than the experts (*p* = 0.068), as in the intact condition. The inversion effect shown by the experts had bigger amplitude than that from the novices (*p* = 0.087). The results were technically not significant enough, but the tendency was clear. It suggested that experts tend to use configural processing to chimpanzee faces, or faces plus blurred body parts, more than the novices. The lack of more significant results could be from the experimental paradigm, or certain expertise for quadrupedal animals of the chimpanzee novices, or both. Different from previous studies^2,12,14,15^, we used zero-delayed matching-to-sample tasks in this study. It fit well for chimpanzee participants, but we cannot rule out the possibilities that it might not be ideal to reveal more subtle differences between two groups of human participants. Another possibility is that the chimpanzee novices, although being novices to chimpanzees, but actually are experts about quadrupedal animals, including cats, dogs, horses, etc. It also suggests that the configural processing of humans can be flexibly tuned or generalized for other objects (species).

This paper also aims to discuss the difference of body processing in the two different species, and both groups of humans showed different features in processing their conspecifics compared to chimpanzees: humans are more sensitive than chimpanzees to body cues.

In this study, we found that humans show the inversion effect for all the conditions of human body stimuli. This suggests that humans used configural processing to intact human bodies, bodies with faces blurred, bodies with all the parts blurred except for faces, bodies without heads, and body silhouettes. It implies that the loss of local details or even faces does not interfere with the configural processing. Chimpanzees, however, rely heavily on the faces and body contours in their configural processing of conspecifics’ bodies, and the inversion effect disappears when the faces are blurred or missing^22^. Although chimpanzees are still able to extract emotional information from body movements with blurred face^26^, for the inversion effect of bodies, they needed clear faces^22^. The difference of chimpanzees and humans implies that, compared to chimpanzees, humans seem to be more sensitive to the general body information to show the inversion effect.

The fact that humans showed the inversion effect to headless bodies is consistent with the finding of Robbins and Coltheart^25^, but contradicts reports from two other studies^7,24^. It tends to support the hypothesis that faces are not necessary to evoke the inversion effect. In fact, the blurring of faces or other body parts does not interfere with configural processing, either. The body contour alone (the silhouette) could evoke the inversion effect. Additionally, in Matsuno and Fujita’s study^17^, it was found that humans showed the inversion effect to intact human bodies, human bodies composed of cylinders and boxes, and body parts. Therefore, the body configural processing of humans might be sensitive to all general body cues, while chimpanzees rely on faces and body contours to a greater extent.

Regarding the expert effect, chimpanzees and humans both use different ways to process the bodies of conspecifics and the bodies of other species. Both are more sensitive to the body cues of conspecifics than they are to the body cues of other species.

The human participants (chimpanzee experts) showed the inversion effect for intact chimpanzee bodies, while the chimpanzees (human experts) did not show the inversion effect for intact human bodies. That is, humans showed the expert effect but chimpanzees did not. This implies that chimpanzees might not have the ability to use configural processing for other species which they have expertise with, such as humans. However, chimpanzees did show the inversion effect for headless human bodies (the body-only condition). Therefore, it could be concluded that chimpanzees are able to partially show the expert effect. In Matsuno and Fujita’s study^17^, it was found that capuchin monkeys showed the inversion effect for intact human bodies, but failed to show it for human bodies composed of cylinders and boxes, or for individual human body parts, both cases where human participants did show the inversion effect. Compared to humans, both chimpanzees and capuchin monkeys are less sensitive to humans’ body cues.

There are several explanations as to why chimpanzees did not show the inversion effect for intact human bodies, yet showed the inversion effect for headless human bodies. One possibility is that they might be slightly afraid to look at human faces directly. Although looking directly is not taken as a threat by chimpanzees, unlike other primate species such as Japanese macaques, chimpanzees prefer not to be looked at directly by humans or to look at humans directly. This has been found to be true of the chimpanzees at the Primate Research Institute. It is probable that the large proportion of white sclera in human eyes accentuates the pupils, which might look intimidating to chimpanzees^27^. Such complex reactions to human faces might have interfered with configural processing in our study. Another possibility is that chimpanzees were very curious ab out the novelty of the humans in the tasks, and they took time examining them. Chimpanzees look at faces longer than other parts of bodies^28^. Therefore, when faces were present, their attention may have been distracted away from the task itself.

Chimpanzees only showed the inversion effect for headless human bodies but not the other four conditions of human bodies. However, they showed the inversion effect for intact chimpanzee bodies, chimpanzee bodies with body parts blurred, and chimpanzee body silhouettes. This suggests that chimpanzees use slightly different ways to process human bodies, and that they are less sensitive to human body cues compared to the bodies of conspecifics, in showing the inversion effect. Human participants demonstrated the same properties. They were very sensitive to human body cues and showed the inversion effect for all five conditions of human bodies, but to chimpanzee bodies, they did not show the inversion effect in the silhouette condition. Although they were all chimpanzee experts, the humans needed more cues, such as the presence of faces or more information about body details, as opposed to the shape of bodies alone (the silhouette chimpanzee condition), to show configural processing for chimpanzee bodies.

Differences exist in how humans process chimpanzee bodies and how chimpanzees process human bodies, but both species find body details important and silhouettes difficult.

When we examine the results of how humans process chimpanzee bodies and how chimpanzees process human bodies, there are two common features: both showed the inversion effect to headless bodies of the other species, and no inversion effect to silhouettes of the other species. This implies that both species need more body clues than a black body contour to evoke the inversion effect for each other. Additionally, humans and chimpanzees use different strategies for processing visual information about one another. This is not an unexpected finding, because they also use different strategies for conspecifics, as shown by the results of this study and the previous one^22^. Concerning the inversion effect to headless bodies by both species, a recent study^29^ found that humans show the inversion effect to headless bodies when they are forward-facing, but no inversion effect was shown to those seen from behind. It suggests that the inversion effect may exist for stimuli in their more familiar forms. In this experiment, all the stimuli were facing towards the participants, and the results were consistent with the findings in this human study.

In Experiment 1, chimpanzees showed *inverted* inversion effects for intact human bodies, human bodies with blurred body parts, and human silhouettes, according to the response-time data. In these conditions, the response times in the upright trials were significantly longer than those in the inverted trials. Farah *et al*.^30^ reported on the inverted face inversion effect in prosopagnosia, and suggested that it implies a neurologically localized module for upright face recognition in humans. Suzuki and Noguchi^31^ reported that unconscious face processing with low-contrast stimuli revealed the reversed face inversion effect in N170 using electroencephalography. In this study, however, it does not seem to fit any of the reported situation. It is possible that the chimpanzees were very curious about the humans, so they examined them by paying special attention to upright faces^28^, and therefore took a longer time in the upright trials than the inverted trials. However, this cannot explain why the chimpanzees showed the inverted inversion effect for human silhouettes. It is possible that upright bodies contain more information than inverted ones, so chimpanzees took more time examining them. In order to solve this problem, a familiarization procedure could be used before testing (e.g.,^32,33^).

Diamond and Carey^2^ concluded that three conditions need to be met to evoke the inversion effect. All the members of the class need to share the same configuration; they should vary in internal relations and be individualized; and subjects must have expertise about them. Bodies, like faces, where the study for configural processing started, meet the first and second criteria apparently. The results of the present study support the third one. Humans show the expert effect for chimpanzees, and as human experts, chimpanzees partially showed the expert effect for human bodies. The performance of human participants is consistent with previous research about expertise with cars, birds, dogs, and other categories^11–13^. Humans are also able to show the expert effect for trained artificial objects called “Greebles”^14–16^. The present study added that not only humans, but also chimpanzees, our closest relatives, have the potential to demonstrate this ability. From an evolutionary perspective, configural processing for bodies and faces might have appeared long before humans, at least for capuchin monkeys^17^ and chimpanzees^22^. During the evolutionary process, this ability might already have had the potential to become more flexibly tuned for objects that might appear later in life but need quick detection and expertise.

In summary, in this study, we used a 2×2 paradigm and compared body processing by chimpanzees and humans for chimpanzee and human bodies, in order to investigate the expert effect by these two species, as well as differences in their approaches to body processing. The human participants were experts on chimpanzees, and the chimpanzee participants were experts on humans. Combined with the findings of our previous study^22^, the results of the present research indicated that humans showed the expert effect, while chimpanzees partially showed it. Both humans and chimpanzees were more sensitive to the body cues of their conspecifics than to those of the other species, which was strongly supported by the absence of the inversion effect in the silhouette condition for the other species. Additionally, humans were more sensitive to humans’ body cues than chimpanzees were to chimpanzees’ body cues in the configural body processing. We then tested chimpanzee novices with chimpanzee body stimuli, although there was not significant differences between chimpanzee novices and experts, we found several tendencies that suggest the effect of expertise on body processing.

## Methods

### Participants

#### Humans that were chimpanzee experts

Nine chimpanzee experts participated in the study (age from 25 to 39 years old, mean 32.56 years old, *SD* = 5.747; female *N* = 7). Eight of them (age from 25 to 39 years old, mean 32.25 years old, *SD* = 6.065; female *N* = 7) finished all the conditions (Experiments 2 and 3), and the remaining one participated in all the conditions with chimpanzee stimuli (Experiment 2) but not those with human stimuli. All of them received the control condition with house stimuli. They were all chimpanzee researchers. The experience with chimpanzees varied from 2 years to 18 years. One participant was experienced in observing chimpanzees in multiple locations, including in captivity and the field. Others mainly worked with the captive chimpanzees at the Primate Research Institute, and all had experience observing wild chimpanzees in the field. All the participants had intensive interactions with the captive chimpanzees on a daily basis for at least 2 years. The research proposal was approved by the Human Research Ethics Committee of the Primate Research Institute, Kyoto University (#2017-11, #2019-13) and all procedure adhered to relative regulations. Informed consent was obtained from all participants.

#### Humans that were chimpanzee novices

Twenty college students from Chubu University participated in the study (age from 18 to 22 years old, mean 20.05 years old, *SD* = 1.432; female *N* = 10). To compare with the experts, these participants were engaged in the chimpanzee body conditions where the experts showed the inversion effect, i.e., the intact body condition, the face-blur condition, the body-blur condition, and the body-only condition. They all had no experience observing or working with non-human primates. The participants were recruited via an internal information system of Chubu University. Students voluntarily registered to join the experiments. Those who joined the study received credit. The choice of not participating was not related to any ratings or gradings to the students. The research proposal was approved by the Human Research Ethics Committee of the Primate Research Institute, Kyoto University (#2019-06), and the Ethics Committee of Chubu University (20190067). All procedures adhered to relative regulations. Informed consent was obtained from all participants.

### Chimpanzees

Seven chimpanzees (The same participants as in our previous study, see 22) participated in the experiment (Table 2). They lived in two social groups of 12 individuals in total at the Primate Research Institute, Kyoto University (Inuyama, Aichi, Japan). All participants were born in captivity except for Ai, who was brought to the institute from the wild when she was about one year old (more details are available in the Great Ape Information Network, see Table 2). Their living environment included an outdoor compound (700 m^2^) and attached indoor facilities^23^. The chimpanzees were free to interact with their group members. The two groups were physically separated from each other, but the living areas are next to each other so they could see and hear the other group members. The group compositions had been changing over the years, and each chimpanzee knew all other chimpanzees very well. The number of individuals had been about 12 from at least the year 2000. Chimpanzees are in upright postures most of the time. The participants see many people every day over the years. They interact intensively with caretakers and researchers every day^23^. They interact intensively with veterinarians and visitors irregularly. They could see people of the research institute and people in the city from their living environment. All chimpanzees had full access to food and water during the experiments. Previously, the chimpanzees had experience with various cognitive tasks using touch screens, including numerical sequence learning, short-term memory tasks, learning tasks of an artificial language, tests about visual attention and visual search, and facial recognition^20,21,34–37^.

**Table 2:** General characteristics of the seven chimpanzees.

Name	GAIN ID Number*	Sex	Age (when the study started)	Kinship	Participated in the previous study**?
Ai	0434	Female	41	Ayumu’s mother	Yes
Ayumu	0608	Male	17	Ai’s son;Pal’s paternal half-sibling	Yes
Chloe	0441	Female	37	Cleo’s mother	Yes
Cleo	0609	Female	17	Chloe’s daughter	Yes
Pan	0440	Female	34	Pal’s mother	No
Pal	0611	Female	17	Pan’s daughter; Ayumu’s paternal half-sibling	Yes
Pendesa	0095	Female	40	N.A.	Yes

Note: *Identification number for each chimpanzee listed in the database of the Great Ape Information Network (GAIN); https://shigen.nig.ac.jp/gain/

**Previous study refers to Gao & Tomonaga (2018).

All procedures adhered to the Japanese Act on the Welfare and Management of Animals. The daily care and use of the chimpanzees adhered to the Third Version of Guidelines for the Care and Use of Laboratory Primates of the Primate Research Institute, Kyoto University. The research proposal was approved by the Animal Research Committee of Kyoto University, and the Animal Welfare and Animal Care Committee of the Primate Research Institute, Kyoto University (#2017-106, #2018-125).

#### Apparatus

**Figure 5:**
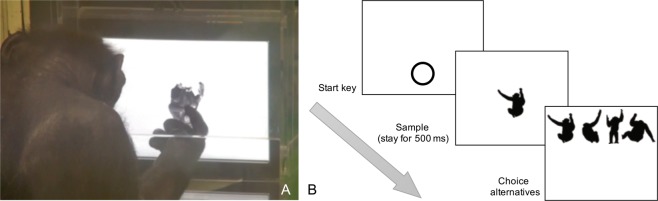
The experiment setting (A) and the general procedure (B). (**A**) Chimpanzee Ai is doing the task. She is touching an inverted sample stimulus. (**B**) In each trial, a start key showed on the screen first. After touching the start key, the participants saw a sample appear on the screen. It would stay for 500 ms and then disapper upon touching. Then, four choice options appeared without any delay. The original pictures of the chimpanzee image on the screen (A) and the silhouette chimpanzee images (B) were provided by Kumamoto Sanctuary, Wildlife Research Center of Kyoto University.

The human participants that were chimpanzee experts and chimpanzee participants used the same apparatus (Fig. 5A). They sat in an experimental booth and operated on a touch screen with a 15-inch LCD display (1,024 × 768 pixels, 1 pixel = 0.297 mm). The screen was not tilted in any direction. The participants faced the screen head-on. During the experiments, the participants could move freely, but they would always sit in front of the screen about 40 cm away. They always kept their postures natural and relaxed, and heads upright. For chimpanzees, when they made a correct choice, they would receive a piece of apple or a raisin delivered by a feeder via a tube, in conjunction with a chime sound. When they made a wrong choice, an error buzzer would sound, and no food was provided. For human participants, a chime or buzzer would sound depending on whether their choice was correct or not. The experimental programs were written and operated with Microsoft Visual Basic 2010 software (Microsoft Corp.; Redmond, WA, USA). The human participants that were chimpanzee novices did the tasks in a lab in Chubu University. They sat on a chair and operated on a touch-screen laptop (10.2-inch LED display, 1,024 × 768 pixels, 1 pixel = 0.203 mm) on the desk in front of them. Other settings were the same as other participants.

#### Stimuli

**Figure 6:**
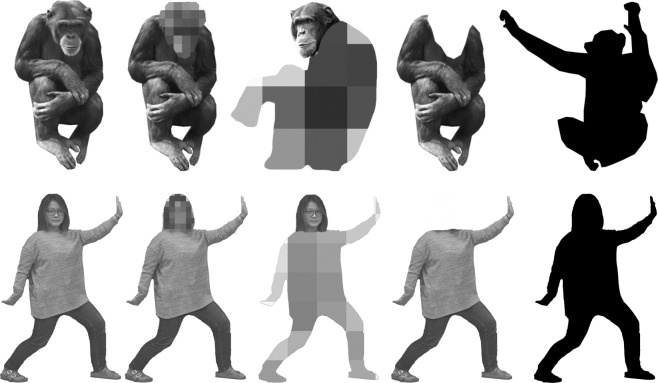
Examples of stimuli. Top row shows chimpanzee stimuli, and bottom row shows human stimuli. From left to right, they are sample stimuli of the intact body condition, the face-blur condition, the body-blur condition, the body-only condition and the silhouette condition. In the face-blur condition, all stimuli had the faces replaced by mosaic patterns, and those in the body-blur condition had the mosaic patterns over the bodies except for the faces. The stimuli in the body-only condition were all headless bodies. Stimuli of human bodies were manipulated in the same manner. The original pictures of the chimpanzee images were provided by Kumamoto Sanctuary, Wildlife Research Center of Kyoto University. The original picture of the human images was taken by the authors.

In this study, the chimpanzees received five conditions of human body stimuli (Experiment 1). The human participants, who were chimpanzee experts received five conditions of chimpanzee body stimuli (Experiment 2) and five conditions of human body stimuli (Experiment 3), which were the same ones used in Experiment 1 (Fig. 6). The human participants that were chimpanzee novices, received chimpanzee body conditions where the experts showed the inversion effect, i.e. the intact body condition, the face-blur condition, the body-blur condition, and the body-only condition (Experiment 4). The chimpanzee body stimuli were identical to the ones used in the same conditions in our previous study^22^. Therefore, direct comparison involving data from the previous study is possible. Additionally, we had a control condition with human participants (the same 9 individuals as in Experiment 2) using stimuli of house pictures.

For each condition, we prepared 20 different body stimuli. Then we inverted these 20 pictures. In total, 40 pictures were used for each condition. All pictures were black and white. They were balanced in luminance. The size of the stimuli was about 400 px × 400 px. In each trial, one stimulus served as the sample, and three other stimuli were randomly chosen from those with the same orientation as distractors. The correct stimuli and their locations were counterbalanced in the experiment. Some pictures were obtained from the Internet, and the rest were provided by Kumamoto Sanctuary, Wildlife Research Center of Kyoto University. The original pictures were manipulated using Adobe Photoshop software (Adobe Systems; San Jose, CA, USA) and Pixelmator (Pixelmator Team Ltd.; Vilnius, Lithuania). We evaluated the similarity of all the pictures (more than the desired number) before choosing those used in the experiment. The participants were not familiar with the specific chimpanzees or humans in the stimulus pictures. The stimuli were all different individuals.

For both chimpanzee and human body stimuli, the manipulations of the five conditions were similar (Fig. 6). 1) Intact conditions: intact chimpanzee or human bodies were used. 2) Face-blur condition: in this condition, all stimuli had their faces replaced with a mosaic pattern, and the rest of the bodies remained intact. 3) Body-blur condition: the bodies were replaced with a mosaic pattern except for the face part. 4) Body-only condition: all the bodies were headless. 5) Silhouette condition: all the bodies were in pure black color, and no details were visible.

## Procedure

The general procedure was zero-delayed matching-to-sample (Fig. 5B). In each trial, a circle, serving as the start key, appeared at the bottom centre of the touch screen against a white background. After touching the start key, a sample stimulus appeared in the centre of the screen. After 500 ms, the sample would disappear upon touching, and without any delay, four choice alternatives in the same orientation as the sample (all upright or all inverted) would appear in the upper part of the screen side by side. If the participants touched the one that was the same as the sample, it was a correct choice. Chimpanzees would receive a piece of apple or raisin as reward along with a chime sound. Human participants would hear the chime sound. If they chose another stimulus other than this one, it was a wrong choice, and a buzzer would sound without any food. The inter-trial-interval for chimpanzees was 1.5 s, and for humans was 0 s. The timeout was 2 s. The upright trials and inverted trials were mixed in each session.

In Experiment 1 (participants: chimpanzees; stimuli: human bodies), each participant received all five conditions. Each condition had eight sessions, and each session had 40 trials. In Experiment 2 (participants: humans that were chimpanzee experts; stimuli: chimpanzee bodies), each participant received all five conditions. Each condition had one session composed of 120 trials. In Experiment 3 (participants: humans that were chimpanzee experts; stimuli: human bodies), each of the eight participants received all five conditions. Each condition had one session of 120 trials. In Experiment 4 (participants: humans that were chimpanzee novices; stimuli: chimpanzee bodies), each of the 20 participants received all four conditions. Each condition had one session of 120 trials. The control condition of house stimuli had the same procedure, with 120 trials of one session for each participant.

## Data analyses

Accuracy and response-time data were analyzed. For each condition, we compared the performances in the upright and inverted trials on a session-by-session basis to examine the inversion effect. We used generalized linear mixed modeling (GLMM) by R 3.4.3^38^ using the “lme4” package^39^. The data points were the average performances in the upright and inverted trials in each session. For chimpanzee response time data, we made justifications to the raw data. Sometimes, unexpected sound occurred outside the experiment area, and chimpanzees would be distracted. For chimpanzees’ response-time data, we first calculated the average and SD within each session, and discarded the trials with the response time longer than average + 3 SD, then we calculated the mean response time again and use this in further analyses. In GLMM, for each condition in each experiment, the dependent variable was accuracy or response time. The fixed effect was orientation (upright or inverted), and the random effects were participant ID and session number (in experiments with humans, there was only one session for each condition, so session number was not included as an effect in GLMM for experiments with humans). Apart from examining the inversion effect in each condition, we did additional analyses to compare the performances of chimpanzee experts and novices in each condition. In these analyses, the dependent variable was accuracy or response time. The fixed effects were orientation (upright or inverted), group (experts or novices), and interaction between orientation and group. The random effect was participant ID. In all analyses, the accuracy data had binomial distribution and the response-time data had gamma distribution.

## Data and materials availability

All data are available in the manuscript and the supplementary materials.

## Supplementary information

Dataset 1.

Supplementary material 2: Additional analyses.
