# Supplementary material for: Body perception in chimpanzees and humans: The expert effect
Source: Sci Rep. 2020 Apr 28;10:7148. doi: 10.1038/s41598-020-63876-x (PMC7189243; doi:10.1038/s41598-020-63876-x)
Supplement: Supplementary file 2 — Supplementary material 2: Additional analyses. [file 41598_2020_63876_MOESM2_ESM.pdf]

Supplementary material 2: Additional analyses

Body perception in chimpanzees and humans: The expert effect

Jie Gao[1, 2, *], Fumito Kawakami[3], Masaki Tomonaga[1]

1: Primate Research Institute, Kyoto University, Inuyama, Aichi, Japan

2: Japan Society for the Promotion of Science, Tokyo, Japan

3: Chubu University, Kasugai, Aichi, Japan

* Correspondence: gao.jie.87c@kyoto-u.jp

Analysis 1: Comparison of the performances of Experiment 1, 2, 3, and 4

In this analysis, we pooled the data of all the conditions of Experiment 1 (participant: chimpanzees; stimuli: human bodies; we call the situation in Experiment 1 "chimp-see-human"), Experiment 2 (participant: humans [chimpanzee experts]; stimuli: chimpanzee bodies; "expert-see-chimp"), Experiment 3 (participant: humans [chimpanzee experts]; stimuli: human bodies; "expert-see-human"), and Experiment 4 (participants: humans [chimpanzee novices]; stimuli: chimpanzee bodies; "novice-see-chimp"). We did GLMM analyses of accuracy and response time, respectively. We used full models. Accuracy had binomial distribution, and response time had gamma distribution. Accuracy and response-time data had the same fixed and random effects in the modelling. The fixed effects were "condition*orientation*experiment", meaning "condition", "orientation", "experiment" and all the possible interactions among the three. "Condition" included intact, face-blur, body-blur, body-only, and silhouette. "Orientation" included upright and inverted. "Experiment" included chimp-see-human, expert-see-chimp, expert-see-human, novice-see-chimp. The random effects were participant ID and session number.

We focused on the interactions with "orientation" to examine whether the differences in upright and inverted trials (inversion effect) were different across situations or not. Table S1 shows the significant interactions (including several

marginally significant results, $p < 0.065$) between orientation and experiment. The column “Condition” shows the basic level of “condition”. The column of “Experiment a” shows the basic level of “experiment”, and “Experiment b” shows the experiment where the inversion effect differs from that in Experiment a. The last two columns show the *p* values in GLMM analyses of accuracy and response-time data, respectively. For example, the result of the first row is that in “intact” condition, the differences in upright and inverted trials, i.e., the inversion effects, were different between chimp-see-human (Experiment 1) and expert-see-chimp (Experiment 2), from both accuracy ($p = 0.030$) and response-time data ($p = 0.001$). Here we did not include those with silhouette condition and novice-see-chimp experiment appearing together, because it does not provide meaningful information as novice-see-chimp experiment did not have a silhouette condition.

Table S1 Significant interactions between “orientation” and “experiment” in Analysis 1[†]

Condition	Experiment a	Experiment b	*p* value (from accuracy data)	*p* value (from response-time data)
Intact	Chimp see human	Expert see chimp	0.030	0.001
	Chimp see human	Expert see human		< 0.001
	Chimp see human	Novice see chimp		< 0.001
Face blur	Chimp see human	Expert see chimp	0.054	0.026
	Chimp see human	Expert see human		0.018
	Chimp see human	Novice see chimp	< 0.001	0.004
	Expert see human	Novice see chimp	0.009	
Body blur	Chimp see human	Expert see chimp	0.007	0.049
	Chimp see human	Expert see human	0.060	< 0.001
	Chimp see human	Novice see chimp		0.019
Body only	Chimp see human	Expert see human	0.003	
	Expert see chimp	Expert see human	0.063	
	Expert see human	Novice see chimp	0.034	
Silhouette	Chimp see human	Expert see human		0.001

† The table included marginally significant results ($p < 0.065$).

Table S2 shows the significant interactions (including marginally significant results, $p < 0.065$) between orientation and condition. The column "Experiment" shows the basic level of "experiment". The column of "Condition a" shows the basic level of "condition", and "Condition b" shows the experiment where the inversion effect differs from that in Condition a. The last two columns show the *p* values in GLMM analyses of accuracy and response-time data, respectively. For example, the result of the first row is that in "chimp-see-human" experiment (Experiment 1), the differences in upright and inverted trials, i.e., the inversion effects, were different between intact and face-blur conditions, from the response-time data ($p = 0.010$). Here we did not include those with silhouette condition and novice-see-chimp experiment appearing together, because it does not provide meaningful information as novice-see-chimp experiment did not have a silhouette condition.

Table S2 Significant interactions between "orientation" and "experiment" in Analysis 1†

Experiment	Condition a	Condition b	*p* value (from accuracy data)	*p* value (from response-time data)
Chimp see human	Intact	Face blur		0.010
	Intact	Body blur	0.016	0.014
	Intact	Body only	0.047	< 0.001
	Intact	Silhouette		0.028
	Face blur	Body only		0.003
	Body blur	Silhouette	0.051	
	Body only	Body blur		0.002
	Body only	Silhouette		0.002
Expert see human	Face blur	Body only	0.017	
Novice see chimp	Intact	Face blur	0.009	
	Face blur	Body blur	0.037	
	Face blur	Body only	0.017	

† The table included marginally significant results ($p < 0.065$).

Table S3 shows the significant 3-way interactions among orientation, condition, and experiment (including marginally significant results, $p < 0.065$). The columns of "Condition a", "Condition b", "Experiment a", and "Experiment b" show the

conditions and experiments for comparison respectively. The last two columns show the *p* values in GLMM analyses of accuracy and response-time data, respectively. For example, the result of the first row is that the differences of the differences in upright and inverted trials in intact and body-only conditions, i.e., the differences of the inversion effects in intact and body-only conditions, were different between chimp-see-human (Experiment 1) and expert-see-chimp (Experiment 2), from the accuracy data ($p = 0.037$). Here we did not include those with silhouette condition and novice-see-chimp experiment appearing together, because it does not provide meaningful information as novice-see-chimp experiment did not have a silhouette condition.

Table S3 Significant 3-way interactions among "orientation", "condition" and "experiment" in Analysis 1†

Condition a	Condition b	Experiment a	Experiment b	*p* value (from accuracy data)	*p* value (from response-time data)
Body only	Intact	Chimp see human	Expert see chimp	0.037	
Body only	Intact	Chimp see human	Expert see human	0.003	
Face blur	Intact	Chimp see human	Novice see chimp	0.028	
Body only	Intact	Chimp see human	Novice see chimp		0.001
Body only	Face blur	Chimp see human	Expert see human	0.041	0.051
Body blur	Face blur	Chimp see human	Novice see chimp	0.018	
Body only	Face blur	Chimp see human	Novice see chimp	0.011	0.047
Body only	Body blur	Chimp see human	Expert see human	0.007	
Silhouette	Body only	Chimp see human	Expert see human	0.010	
Face blur	Body only	Expert see chimp	Expert see human	0.019	
Intact	Body only	Expert see chimp	Expert see human	0.065	

† The table included marginally significant results ($p < 0.065$).

Analysis 2: Comparison of the performances of Experiment 1, 2, 3, and conditions from Gao & Tomonaga (2018)

In the last analysis, we compared the performances across the experiments and conditions. In this analysis, we included data from Gao & Tomonaga (2018) to

compare the performances across participant species, stimulus species, and conditions. The data we used from Gao & Tomonaga (2018) were conditions of intact chimpanzee bodies ("Experiment 1b" in that study), face-blur, body blur, body-only and silhouette chimpanzee bodies. They are the same five conditions that appeared in Experiment 1, 2, and 3 in this study. The participant species were chimpanzees in Experiment 1 and the cited study, and humans in Experiment 2 and 3, respectively. The stimulus species were humans in Experiment 1 and 3 of this study, and chimpanzees in Experiment 2 of this study and the cited study, respectively. Therefore, we could break the factor "experiment" into "participant species" and "stimulus species". We did GLMM analyses of accuracy and response time, respectively. We used full models. Accuracy had binomial distribution, and response time had gamma distribution. Accuracy and response-time data had the same fixed and random effects in the modelling. The fixed effects were "condition*orientation*participant species*stimulus species", meaning "condition", "orientation", "participant species", "stimulus species" and all the possible interactions among the four. "Condition" included intact, face-blur, body-blur, body-only, and silhouette. "Orientation" included upright and inverted. "Participant species" and "stimulus species" both included chimpanzees and humans. The random effects were participant ID and session number.

We focused on the interactions with "orientation" to examine whether the differences in upright and inverted trials (inversion effect) were different across situations or not. Table S4 shows the significant interactions (including marginally significant results, $p < 0.065$) between orientation and condition. The columns "Participant" and "Stimuli" show the basic level of "experiment" and "stimuli" respectively. The column of "Condition a" shows the basic level of "condition", and "Condition b" shows the experiment where the inversion effect differs from that in Condition a. The last two columns show the *p* values in GLMM analyses of accuracy and response-time data, respectively. For example, the result of the first row is that when participants were chimpanzees and when stimuli were chimpanzee bodies, the

differences in upright and inverted trials, i.e., the inversion effects, were different between intact and face-blur conditions, from the accuracy data ($p < 0.001$).

Table S4 Significant interactions between “orientation” and “condition” in Analysis 2†

Participant	Stimuli	Condition a	Condition b	*p* value (from accuracy data)	*p* value (from response-time data)
chimpanzee	chimpanzee	intact	face blur	< 0.001	
			body blur	0.063	
			body only	< 0.001	
			silhouette	0.014	
		face blur	body blur	0.052	
		body blur	body only	0.002	
		body only	silhouette	0.014	
chimpanzee	human	intact	face blur		0.022
			body blur	0.016	0.028
			body only	0.048	< 0.001
			silhouette		0.049
		face blur	body only		0.008
		body blur	body only		0.006
			silhouette	0.052	
		body only	silhouette		0.005
human	human	face blur	body only	0.017	

† The table included marginally significant results ($p < 0.065$).

Table S5 shows the significant interactions (including marginally significant results, $p < 0.065$) between orientation and participant. The columns “Stimuli” and “Condition” show the basic level of “stimuli” and “condition” respectively. The column of “Participant a” shows the basic level of “participant species”, and “Participant b” shows the experiment where the inversion effect differ from that in Participant a. The last two columns show the *p* values in GLMM analyses of accuracy and response-time data, respectively. For example, the result of the first row is that in when stimuli were chimpanzee bodies and when condition was body-blur, the differences in upright and inverted trials, i.e., the inversion effects, were different between human and chimpanzee participants, from the accuracy data ($p = 0.027$).

Table S5 Significant interactions between "orientation" and "participant" in Analysis 2†

Stimuli	Condition	Participant a	Participant b	*p* value (from accuracy data)	*p* value (from response-time data)
chimpanzee	body blur	chimpanzee	human	0.027	
human	intact	chimpanzee	human		0.001
	face blur	chimpanzee	human		0.039
	body blur	chimpanzee	human	0.059	0.004
	body only	chimpanzee	human	0.003	
	silhouette	chimpanzee	human		0.005

† The table included marginally significant results ($p < 0.065$).

Table S6 shows the significant interactions (including marginally significant results, $p < 0.065$) between orientation and stimuli. The columns "Participant" and "Condition" show the basic level of "participant species" and "condition" respectively. The column of "Stimulus a" shows the basic level of "stimuli", and "Stimulus b" show the experiment where the inversion effect differ from that in Stimulus a. The last two columns show the *p* values in GLMM analyses of accuracy and response-time data, respectively. For example, the result of the first row is that in when participants were chimpanzees and when condition was intact, the differences in upright and inverted trials, i.e., the inversion effects, were different between human and chimpanzee stimuli, from both the accuracy data ($p < 0.001$) and response-time data ($p = 0.028$).

Table S6 Significant interactions between "orientation" and "stimuli" in Analysis 2†

Participant	Condition	Stimulus a	Stimulus b	*p* value (from accuracy data)	*p* value (from response-time data)
chimpanzee	intact	chimpanzee	human	< 0.001	0.028
	face blur	chimpanzee	human		0.039
	body blur	chimpanzee	human		0.036
	silhouette	chimpanzee	human	0.017	
human	body only	chimpanzee	human	0.063	

† The table included marginally significant results ($p < 0.065$).

Table S7 shows the significant 3-way interactions among orientation, condition, and participant species. The column of “Stimuli” shows the basic level of stimuli. In this table they are all human bodies. The columns of “Participant a”, “Participant b”, “Condition a”, and “Condition b” show the participant species and conditions for comparison respectively. The last two columns show the *p* values in GLMM analyses of accuracy and response-time data, respectively. For example, the result of the first row is that when stimuli were human bodies, the differences of the differences in upright and inverted trials in intact and body-only conditions, i.e., the differences of the inversion effects in intact and body-only conditions, were different between chimpanzee and human participants, from the response-time data (p = 0.009).

Table S7 Significant 3-way interactions among “orientation”, “condition” and “participant species” in Analysis 2

Stimuli	Participant a	Participant b	Condition a	Condition b	*p* value (from accuracy data)	*p* value (from response-time data)
human	chimpanzee	human	intact	body only		0.009
	chimpanzee	human	face blur	body only	0.040	0.019
	chimpanzee	human	body blur	body only		0.019
	chimpanzee	human	body only	silhouette		0.023

Table S8 shows the significant 3-way interactions among orientation, condition, and stimulus species (including marginally significant results, p < 0.065). The column of “Participant” shows the basic level of participant. In this table they are all human bodies. The columns of “Stimulus a”, “Stimulus b”, “Condition a”, and “Condition b” show the stimulus species and conditions for comparison respectively. The last two columns show the *p* values in GLMM analyses of accuracy and response-time data, respectively. For example, the result of the first row is that when participants were chimpanzees, the differences of the differences in upright and inverted trials in intact and face-blur conditions, i.e., the differences of the inversion effects in intact and

face-blur conditions, were different between chimpanzee and human stimuli, from the accuracy data ($p < 0.001$).

Table S8 Significant 3-way interactions among “orientation”, “condition” and “stimulus species” in Analysis 2†

Participant	Stimulus a	Stimulus b	Condition a	Condition b	*p* value (from accuracy data)	*p* value (from response-time data)
chimpanzee	chimpanzee	human	intact	face blur	< 0.001	
				body blur	0.003	
				body only	< 0.001	0.009
				silhouette	0.028	
			face blur	body only		0.037
			body blur	body only	0.038	0.011
			body only	silhouette	0.004	0.041
human	chimpanzee	human	intact	body only	0.065	
			face blur	body only	0.019	

† The table included marginally significant results ($p < 0.065$).

About the 4-way interactions of orientation, participant species, stimulus species, and condition, we did not find significant ones, but there were two marginally significant 4-way interactions. The differences of the differences of the differences in upright and inverted trials in body-only and face-blur conditions in chimpanzee and human participants, i.e., the differences of the differences of the inversion effects in body-only and face-blur conditions in chimpanzee and human participants, were different between chimpanzee and human stimuli, from the accuracy data ($p = 0.059$). The differences of the differences of the differences in upright and inverted trials in body-only and body-blur conditions in chimpanzee and human participants, i.e., the differences of the differences of the inversion effects in body-only and body-blur conditions in chimpanzee and human participants, were different between chimpanzee and human stimuli, from the response-time data ($p = 0.058$).

References

[1] Gao, J., & Tomonaga, M. The body inversion effect in chimpanzees (*Pan troglodytes*). *PLOS ONE.* **13**, e0204131 (2018).
